# Revisited Upper Reference Limits for Highly Sensitive Cardiac Troponin T in Relation to Age, Sex, and Renal Function

**DOI:** 10.3390/jcm10235508

**Published:** 2021-11-25

**Authors:** Christiane Gärtner, Romy Langhammer, Maria Schmidt, Martin Federbusch, Kerstin Wirkner, Markus Löffler, Berend Isermann, Ulrich Laufs, Rolf Wachter, Thorsten Kaiser

**Affiliations:** 1 Institute for Laboratory Medicine, Clinical Chemistry and Molecular Diagnostics, University of Leipzig Medical Center and Medical Faculty, 04109 Leipzig, Germany; christiane.gaertner@medizin.uni-leipzig.de (C.G.); Maria.Schmidt@medizin.uni-leipzig.de (M.S.); martin.federbusch@medizin.uni-leipzig.de (M.F.); berend.isermann@medizin.uni-leipzig.deleipzig.de (B.I.); 2 Clinic and Polyclinic for Cardiology, University of Leipzig Medical Center, 04103 Leipzig, Germany; Romy.Langhammer@medizin.uni-leipzig.de (R.L.); ulrich.laufs@medizin.uni-leipzig.de (U.L.); rolf.wachter@medizin.uni-leipzig.de (R.W.); 3 LIFE—Leipzig Research Center for Civilization Diseases, University of Leipzig, 04109 Leipzig, Germany; Kerstin.Wirkner@life.uni-leipzig.de (K.W.); markus.loeffler@imise.uni-leipzig.de (M.L.); 4 Institute for Medical Informatics, Statistics, and Epidemiology (IMISE), University of Leipzig, 04109 Leipzig, Germany

**Keywords:** troponin, cardiac markers, reference intervals

## Abstract

(1) Background: Highly sensitive cardiac troponin T (hs-cTnT) plays an essential role in the diagnosis of myocardial injury. The upper reference limit of the respective assay is generally applied, irrespective of age, renal function, or sex. We aimed to identify age-adjusted and sex-adjusted upper reference limits in relation to renal function in a large population-based cohort without cardiac diseases. (2) Methods: We included 5428 subjects of the population-based LIFE-Adult cohort, free of diagnosed cardiac diseases. Sex-adjusted and age-adjusted 99th percentiles for hs-cTnT in subjects with preserved renal function were obtained. (3) Results: The hs-cTnT values were higher in men of all age groups. In both sexes, an increasing age positively correlated with higher hs-cTnT values. Hs-cTnT weakly correlated with serum creatinine. The three-dimensional analysis of age, creatinine, and hs-cTnT showed no relevant additional effect of creatinine on hs-cTnT. In men aged above 60 and women above 70, the calculated 99th percentiles clearly exceeded the commonly applied thresholds. (4) Conclusion: Age and sex have a major impact on the serum concentration of hs-cTnT, while renal function does not. We propose to consider age-adjusted and sex-adjusted reference values.

## 1. Introduction

Highly sensitive cardiac troponin T (hs-cTnT) has evolved into an essential diagnostic tool to detect myocardial injury—especially in patients presenting with acute chest pain. By definition, troponin T values above the 99th percentile compared to a healthy reference population are declared elevated [1,2,3]. For the widely used Elecsys^®^ fifth generation hs-cTnT assay developed by Roche Diagnostics, the upper reference limit of 14 ng/L was derived and confirmed [4,5]. This 99th percentile is generally used as an upper reference limit concentration for delineation in rule-in and rule-out algorithms according to the currently used guidelines for the management of patients presenting with acute coronary syndromes without persistent ST-segment elevation [1,2].

Nonetheless, elevated hs-cTnT levels are regularly found in asymptomatic patients as well and may cause diagnostic uncertainties. Independent of cardiac diseases, there are several factors that may influence the serum concentration of hs-cTnT, for example, age, sex, and kidney function. The currently used upper reference limits do not consider these factors, although relevant effects on hs-cTnT have been reported in multiple studies.

The upper reference limit of 14 ng/L for the Elecsys^®^ assay (Roche Diagnostics, Indianapolis, IN, USA) was derived from a population of 616 “apparently healthy volunteers” with a mean age of 44 years [4]. However, the majority of patients presenting with acute chest pain or other heart-related complaints are considerably older. Several community-based studies have suggested an adjusted upper reference limit of >35 ng/L for cardiac-healthy males and >25 ng/L for cardiac-healthy females over 65 years [6,7,8]. The 99th percentile values rise even further with increasing age [6,8,9].

A patient’s sex may also have a major influence on the hs-cTnT serum concentration. Several studies in different countries have shown significantly higher values in men, which is assumed to be a result of higher cardiac muscle mass on average [4,6,7,10,11,12,13,14,15,16]. The 99th percentile of hs-cTnT values in women aged <65 years was consistently below the commonly applied threshold of 14 ng/L [4,6,7,11,12,13,14].

Troponin T levels also rise with declining renal function measured by eGFR due to multiple reasons, such as a reduced renal elimination and myocardial injury [17,18,19,20,21,22,23,24,25]. Still, we lack specific hs-cTnT upper reference limits for different stages of renal insufficiency.

In summary, there is a clear need for hs-cTnT reference values stratified by age, sex, and renal function. The interpretation of hs-cTnT values in the clinical setting using the current fixed threshold of 14 ng/L (for the Elecsys^®^ hs-cTnT assay) may result in suboptimal diagnostic decision making, e.g., underdiagnosing cardiac diseases in women under 65 years or overdiagnosing older or renal-insufficient patients. We aim to address this critical knowledge gap in the present study by identifying hs-cTnT reference values adjusted to a person’s age, sex, and renal function in a large community-based, cardiac-healthy population.

## 2. Materials and Methods

### 2.1. Study Population

**Figure 1:**
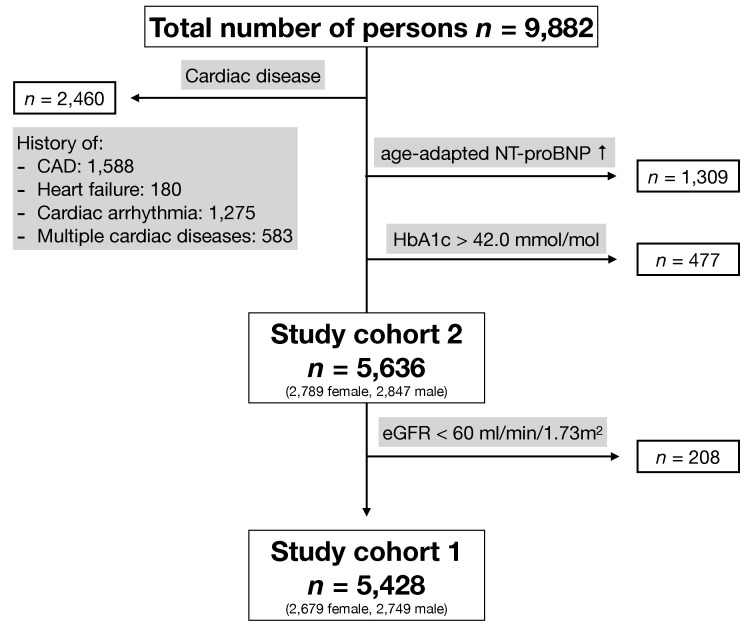
Study population: of 9882 adults, 2460 were excluded due to cardiac diseases, and 1786 were excluded due to abnormal NT-proBNP or HbA1c values. A total of 5636 participants were included in the analysis of hs-cTnT values in relation to sex and renal function (study cohort 2). For the calculation of age-adjusted and sex-adjusted hs-cTnT values, participants with an impaired eGFR were excluded (study cohort 1). CAD: coronary artery disease; HbA1c: hemoglobin A1c; NT-proBNP: amino-terminus pro B-type natriuretic peptide; eGFR: estimated glomerular filtration rate.

In this retrospective study, 9882 adults of the LIFE-Adult cohort were enrolled. LIFE-Adult is a population-based study cohort of 10,000 mainly white individuals living in the city of Leipzig, Saxony (Germany) [26]. Participants were enrolled between 2011 and 2014 by random sampling from the register of residents. Only participants with available hs-cTnT values were included in our study. For the calculation of hs-cTnT upper reference limits, defined as the 99th percentile of a healthy population, we further excluded persons with diagnosed or suspected cardiac disease; these individuals were identified via questionnaire and laboratory diagnostics. If the existence of an underlying heart disease was confirmed or typical angina pectoris was reported in the questionnaire, the person was not included for further analysis. Guided by current recommendations [27], we further excluded individuals with abnormal values in at least one of the following laboratory parameters: amino-terminus pro B-type natriuretic peptide (NT-proBNP) > 125 ng/L for subjects < 5 years and >450 ng/L for subjects ≥ 75 years and hemoglobin A1c (HbA1c) > 42.0 mmol/mol (6.0%). For the calculation of age-adjusted and sex-adjusted upper reference limits, individuals with an estimated glomerular filtration rate (eGFR) of <60 mL/min/1.73 m^2^ were excluded as well (Figure 1).

### 2.2. Laboratory Measurements

Laboratory tests were performed in an accredited laboratory (ISO 17025 and 15189) on fresh biospecimen on the day of sample collection. The serum hs-cTnT and NT-proBNP analyses were performed by using the electrochemiluminescence immunoassay (ECLIA) “Troponin T hs” and “proBNP II” on a cobas® 8000 (e 602, Roche Diagnostics, Mannheim, Germany). Hs-cTnT levels of <3 ng/L were set as 3 ng/L in our analysis. The HbA1c measurements were conducted by turbidimetric inhibition immunoassay (TINIA, A1C-3/Tina-quant Hemoglobin A1c Gen.3, cobas^®^ 8000, c 502, Roche Diagnostics). We used colorimetric enzymatic assays to obtain serum creatinine levels (CREPs2/Creatinine plus ver.2, cobas^®^ 8000, c 502, Roche Diagnostics). The eGFR was calculated on the basis of serum creatinine concentration by using the chronic kidney disease epidemiology collaboration (CKD-EPI) equation [28], which most accurately correlates with measured GFR in our predominantly non-black cohort [29]. All assays have been performed according to the manufacturer’s instructions.

### 2.3. Statistical Analysis

We used Spearman’s rank correlation for correlation analyses of sex-specific hs-cTnT concentration and either age, creatinine level, or eGFR, respectively. Differences with a *p*-value of <0.05 were defined as statistically significant. The 99th percentile and median values were calculated with NumPy’s percentile function [30]. The 95% confidence intervals for percentiles were calculated with the exact method without interpolation as implemented in the textttquantileCI R package [31]. We first ascertained gender-specific reference values for the age groups 20–40, 41–50, 51–60, 61–70, and 71–80 years in patients with sufficient renal function (eGFR ≥ 60 mL/min/1.73 m${}^{2}$). For the continuous graphical representation, floating medians and 99th percentiles were calculated for each year of age with a window size of +/−5 years. In the second step, we conducted gender-specific hs-cTnT medians for different ranges of serum creatinine (0–50, 51–100, 101–150, 151–200, and 201–250 μmol/L). The same was performed for different stages of renal function by means of the following eGFR ranges: 121–150, 91–120, 61–90, 31–60, and ≤30 mL/min/1.73 m${}^{2}$. The continuous graphical representation of sex-specific hs-cTnT floating median values against creatinine (in increments of 10 μmol/L) and eGFR (in increments of 3 mL/min/1.73 m${}^{2}$) was performed similarly to the age analysis, including the values of a +/−15 μmol/L and +/−10 mL/min/1.73 m${}^{2}$ range per point, respectively. In the last step, we conducted a graphical three-dimensional analysis of hs-cTnT values depending on age and renal function for each sex separately.

Statistical analyses were performed with the statistical computing tool R 3.6.2 (The R Foundation for Statistical Computing, Vienna, Austria) [32]. Plots were created by using R’s ggplot2 library and rgl [33,34]. For parsing and filtering of the data, we used the software Python 3.7 (Python Software Foundation, Beaverton, OR, USA) [35].

## 3. Results

### 3.1. Baseline Characteristics

Of the 9882 participants with available hs-cTnT values, 4246 were excluded due to elevated biomarkers suggestive of cardiovascular disease, diabetes, a history of cardiac disease, or intermittent angina pectoris. A total of 5636 participants were included in the analysis of hs-cTnT values in relation to age, sex, and kidney function (study cohort 2, Figure 1). For the calculation of age-adjusted and sex-adjusted upper reference limits of participants with preserved kidney function, an additional 208 individuals were excluded due to renal impairment, leaving a total of 5428 subjects (study cohort 1, Figure 1). The baseline demographic and laboratory characteristics of both study cohorts are presented in Table 1.

### 3.2. Hs-cTnT Upper Reference Limits in Relation to Age and Sex

**Figure 2:**
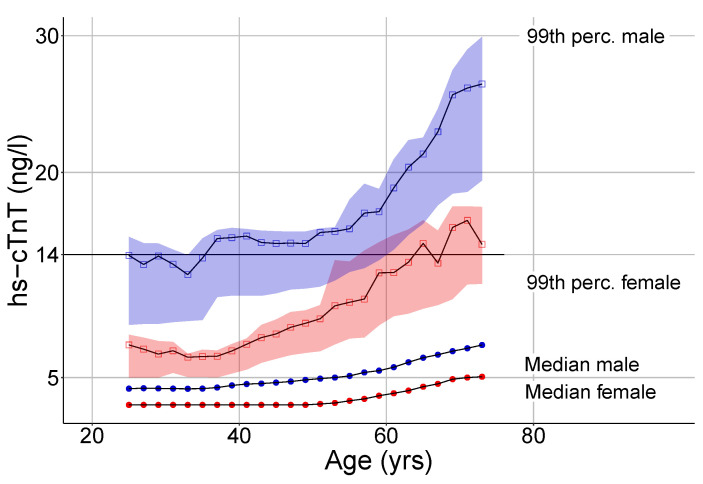
Age-specific floating median (circles) and 99th percentile (squares) hs-cTnT values in females (red) and males (blue). The ranges of 97th–99.4th hs-cTnT percentiles (smoothed with a window size of +/−10 years per point) are highlighted in light red (females) and light blue (males). Hs-cTnT: highly sensitive troponin T.

We calculated lower median and 99th percentile values of hs-cTnT in female subjects compared to males throughout all age groups in study cohort 1 (Table 2, Figure 2). Among both men and women, hs-cTnT levels positively correlated with age (ρ = 0.42, *p* < 0.0001). Figure 2 displays the floating median values and 99th percentiles against age for both sexes. In men, floating median hs-cTnT values are stable until the age of 50 and showed a continuous increase thereafter. An hs-cTnT level of 14 ng/L was confirmed as the 99th percentile threshold for men up to 60 years of age. The calculated 99th percentile curve rises with further increasing age, reaching a maximum of 30.1 ng/L in participants aged 71–80.

Median and 99th percentile hs-cTnT blood concentrations in women do not reach those of men until approximately 10 years later. Women below approximately 60 years of age have 99th percentile values clearly below the defined threshold of 14 ng/L. The curve crosses the threshold at the age of 62 years and reaches a maximum of 17.8 ng/L in the oldest individuals enrolled (71–80 years).

Age-adjusted and sex-adjusted upper reference limits for hs-cTnT were calculated for patients with a preserved eGFR of ≥60 mL/min/1.73 m${}^{2}$ (study cohort 1). The computed median and 99th percentile hs-cTnT values per age group as well as the corresponding number of participants are displayed in Table 2 for both sexes.

### 3.3. Influence of Renal Function on hs-cTnT Values

**Figure 3:**
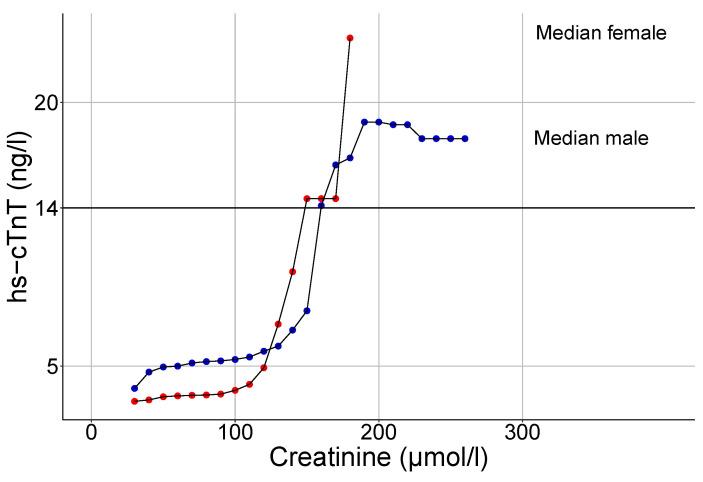
Floating median hs-cTnT values in relation to serum creatinine (window size of +/−15 μmol/L per point, steps of 10 μmol/L) in females (red curve) and males (blue curve). Increasing serum creatinine values correlate with higher median hs-cTnT concentrations. Hs-cTnT: highly sensitive troponin T.

We found a weak positive correlation of hs-cTnT levels and creatinine in study cohort 2 (ρ = 0.16 in women and 0.18 in men, *p* < 0.0001 in both). The graphic representation of the median hs-cTnT concentration against the serum creatinine level in Figure 3 shows increased hs-cTnT values with rising creatinine levels (indicating falling renal function) in both sexes. In females, there is a steeper increase in hs-cTnT values at serum creatinine concentrations above 100 μmol/L. The median hs-cTnT curve crosses the upper reference limit of Roche’s Elecsys^®^ hs-cTnT assay (14 ng/L) at an approximate creatinine concentration of 150 μmol/L in both sexes.

For the calculation of median hs-cTnT values adjusted to renal function, subjects were subdivided by sex and grouped by serum creatinine (in bins of 50 μmol/L). Most subjects had serum creatinine values between 51 and 100 μmol/L (Table 3) with a low number of female subjects with a creatinine concentration above 100 μmol/L and male subjects with a creatinine level above 150 μmol/L. Guided by current expert recommendations [27], the calculation of the 99th percentile values were restricted to age groups of at least 300 individuals per sex. Increasing serum creatinine values are associated with higher median hs-cTnT values as well as higher participant ages.

**Figure 4:**
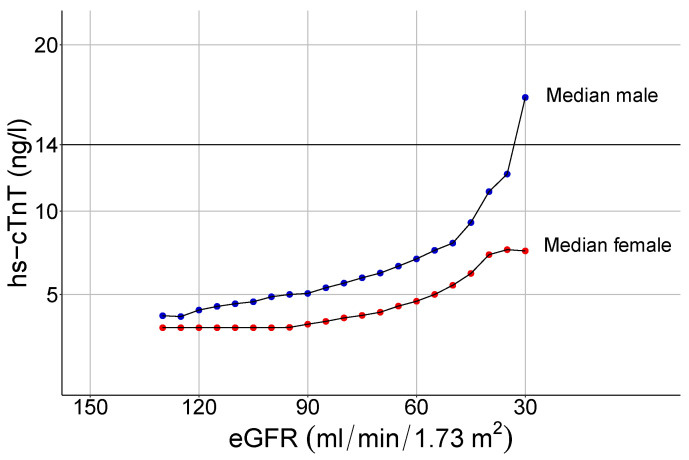
Floating median hs-cTnT values in relation to eGFR (window size of +/−10 mL/min/ 1.73 m^2^ per point, steps of 3 mL/min/1.73 m^2^) in females (red curve) and males (blue curve). There is a negative correlation between eGFR and hs-cTnT concentrations. Hs-cTnT: highly sensitive troponin T; eGFR: estimated glomerular filtration rate.

We next calculated the median hs-cTnT concentration according to sex and eGFR (renal function parameter based on age, sex, and serum creatinine) in study cohort 2 (see Table 4). Hs-cTnT levels and eGFR negatively correlated with medium strength (ρ = −0.32, *p* < 0.0001). Similarly to serum creatinine, the hs-cTnT values increase with falling renal function, characterized by decreasing eGFR (Figure 4).

### 3.4. Hs-cTnT Levels in Relation to Age, Sex, and Renal Function—A Three-Dimensional Analysis

**Figure 5:**
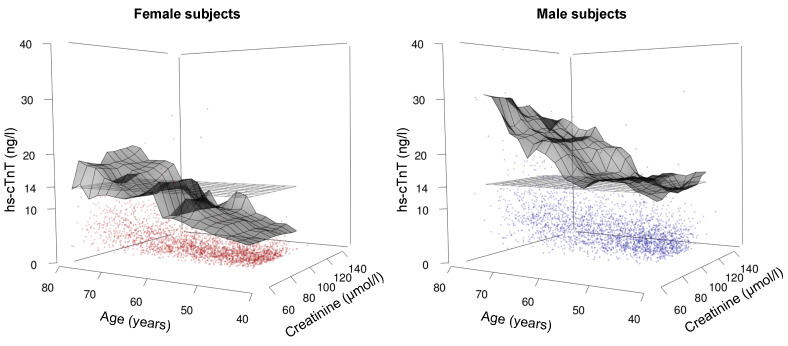
Three-dimensional illustration of hs-cTnT values in relation to age and serum creatinine in study cohort 2 in females (left, red dots) and males (right, blue dots). Floating 99th percentiles are displayed as a grey surface area. In addition to age, serum creatinine concentrations show no discernible added effect on hs-cTnT percentiles. Hs-cTnT: highly sensitive troponin T.

The final aim of this study was to illustrate the combined influence of all three parameters, age, sex, and renal function, on hs-cTnT values. Due to the small number of participants with moderately or highly impaired renal function in study cohort 2, we limited this three-dimensional analysis to graphical exploration. A surface of the 99th percentile hs-cTnT values for combinations of creatinine and age was plotted separately for each sex (Figure 5). Remarkably, serum creatinine concentrations showed an almost negligible additional effect on hs-cTnT percentiles in addition to age.

## 4. Discussion

The key finding of this study is that currently applied hs-cTnT reference values are only valid for men up to the age of 60 and women aged 60–70. We propose using age-adjusted and sex-adjusted reference values, especially in elderly people.

### 4.1. The Impact of Sex on Hs-cTnT Plasma Levels

Our finding of lower hs-cTnT concentrations in women than in men corroborates previous findings [4,6,7,10,11,12,13,14]. This may result in the undertreatment of myocardial infarction in women [36]. However, the use of sex-adjusted troponin upper reference limits is controversial. Available studies show an inconsistent picture of the clinical superiority of sex-adjusted troponin upper reference limits in patients with suspected acute coronary syndrome [37]. Shah et al. demonstrated similar proportions of men and women diagnosed with type 1 myocardial infarction after the implementation of sex-specific upper reference limits for highly sensitive cardiac troponin I [36]. Similar results were reported for hs-cTnT levels in a sub-analysis of the TRAPID-AMI trial [38]. In contrast, Rubini Giménez et al. did not find any benefit of sex-related hs-cTnT upper reference limits in diagnosing AMI [39]. However, the median age of their female participants was 68 years; thus, the lack of additional AMI diagnoses might have been due to an additional age-related effect (the upper reference limit for women aged 68 was approximately 14 ng/L in our study, see Figure 2).

In summary, heart-related complaints are likely to be underdiagnosed in women using uniform hs-cTnT reference values. Therefore, expert associations such as the Academy of the American Association for Clinical Chemistry (AACC) and the International Federation of Clinical Chemistry and Laboratory Medicine (IFCC) endorse the determination of sex-specific hs-cTnT upper reference limits ascertained in a defined, cardiac-healthy reference population [27]. First attempts to define such gender-specific hs-cTnT 99th percentiles in a well-phenotyped and appropriately sized reference population were made by Gore et al. [6]. Our results confirm those thresholds approximately for a European cohort and with a higher number of male patients aged >75 years.

### 4.2. The Impact of Age on Hs-cTnT Plasma Levels

A positive correlation between age and hs-cTnT concentration has been reported previously and was validated by our data. A unique feature of our study is the proposal of age-specific and sex-specific hs-cTnT upper reference limits in 10-year intervals for people aged 20–80 in an adequately sized cohort of assumably cardiac healthy people. Our results indicate that the upper reference limit of 14 ng/L for hs-cTnT (Elecsys^®^ assay) does not suffice in men aged above 60 and women older than 70 years, whereas in women below 60 years this threshold is too high. Only a few studies focused on defining hs-cTnT concentration upper reference limits in different age groups and with particular attention on the elderly population. Gore et al. retrospectively analyzed three US cohorts regarding hs-cTnT 99th percentiles in the age groups of <50, 50–64, 65–74, and ≥75 years [6]. We obtained similar upper reference limits in people up to 65 years. In older age groups, Gore et al. described higher 99th percentiles. This might be due to racially more heterogenous study cohorts with a high percentage of Black individuals (20.6–41.5%; our study population was predominantly white), as the authors generally reported higher hs-cTnT values in Black versus non-Black subjects. Kuster et al. focused on hs-cTnT levels in elderly people. They prospectively analyzed 591 French, ambulatory geriatric individuals (35% male), of which 186 had no diagnosed cardiac or renal disease [8]. Serum hs-cTnT 99th percentiles were predicted for six age groups from 65 to 90 years. Again, increased hs-cTnT values appeared with rising age, but a precise calculation of the 99th percentiles was limited by the small sample size.

In summary, there remains a knowledge gap for hs-cTnT reference values in elderly people. Our study fills this gap for the age group of 71–80 years. Further studies are necessary for identifying additional reference values for individuals > 80 years.

### 4.3. The Impact of Renal Function on Hs-cTnT Plasma Levels

A reference population for the identification of hs-cTnT upper reference limits is commonly defined as being free of kidney disease [27]. Still, due to the high comorbidity of (coronary) heart and chronic kidney disease, clinicians are regularly faced with elevated hs-cTnT values in patients with renal impairment. Myocardial injury may initially occur due to inflammation processes in chronic renal failure patients. Secondly, chronic kidney insufficiency is frequently caused by vascular pathologies and is, therefore, associated with a higher incidence of cardiovascular diseases. Multiple studies have reported an association between impaired renal function and increased troponin T serum concentration [17,18,19,20,21,23]. Those analyses mainly focused on the relationship between hs-cTnT level and eGFR. In contrast, we used serum creatinine as a marker of renal function, since eGFR is directly calculated from age and sex. Here, we found a weak positive correlation between serum hs-cTnT and creatinine levels with a negligible additional effect on hs-cTnT percentiles. The weak correlation between serum hs-cTnT and creatinine levels, as well as the previously described indirect correlation of hs-cTnT and eGFR, seem to be an age-related effect primarily. The fact that in our study cohort females showed higher median hs-cTnT values in participants with a creatinine above 100 µmol/L than men can be explained by the higher age of those subjects on the one hand and a low number in this group on the other hand (Table 3). As our study cohort only included a few participants with advanced kidney disease, the relationship between serum creatinine and hs-cTnT levels (after elimination of the potential age effect) should be the subject of future studies with highly specific study cohorts, which—in case of a significant association—allow a thorough calculation of serum creatinine-adjusted hs-cTnT 99th percentile values.

### 4.4. Limitations

All results were conducted retrospectively and require further prospective validation. Additional limitations lie in the recruitment of the reference population. We defined participants as “cardiac healthy” based on their medical history, current symptoms, and clinical chemistry (including eGFR, NT-proBNP, and HbA1c levels). A morphological evaluation including echocardiography or cardiac magnetic resonance tomography was not performed. The AACC and IFCC task forces outlined that higher upper reference limits for hs-cTnT appear if only health questionnaires are used to identify a presumably cardiac-healthy reference population [27]. However, Giannitsis et al. calculated similar gender-specific reference values for hs-cTnT in cohorts with and without additional cardiac imaging [14]. Therefore, the selection criteria of our study population appear suitable. The fact that our results are similar or lower compared to previously reported upper reference limits of more precisely characterized populations might also be due to a positive selection of participants with a high social status and healthier lifestyle in the LIFE cohort [40]. Additionally, nearly all participants were white; thus, we could not describe the race-specific differences previously demonstrated by others [6].

A final limitation lies in the cutoff value for the eGFR (≥60 mL/min/1.73 m^2^), applied in the selection process of our study cohort. This cutoff has also been used in other studies recently [4,6,7]. An eGFR of 60–89 mL/min/1.73 m^2^ defines a “mildly reduced” kidney function, as observed in the current guidelines [41]. Thus, “normal” kidney function is assumed at eGFR ≥ 90 mL/min/1.73 m^2^. In our opinion, using this higher and stricter cutoff for the selection of our cohort would be unrealistic in the elderly, as kidney function is well known to decline with age (Supplementary Materials, Figure S1). Despite the suspected positive selection of our cohort, 96.3% of the female and 97.7% of the male participants aged 70–80 would have been excluded due to an “impaired kidney function”, although an eGFR between 60 and 90 mL/min/1.73 m^2^ is common at this age. A calculation of age-adjusted median and 99th percentile hs-cTnT values in participants with an eGFR ≥ 90 mL/min/1.73 m^2^ is presented in Supplementary Materials (Supplementary Table S1; Supplementary Figures S2 and S3). The more stringent eGFR cutoff did not relevantly affect median and 99th percentile hs-cTnT values in participants below the age of 60, while the elderly population above 60 years was not sufficiently represented.

## 5. Conclusions

Our results emphasize the relevant interaction of hs-cTnT level with age and sex, while the effect of renal impairment is only of limited relevance for patients with a creatinine value of <150 µmol/L. Further studies are needed for patients with a higher grade of renal insufficiency. Hs-cTnT values exceeding the commonly applied upper reference limit in patients not suspected of acute myocardial infarction cause uncertainties and further diagnostic procedures such as consulting a cardiologist, echocardiography, or coronary angiography. This may result in overdiagnosis due to an inappropriate understanding of which hs-cTnT concentrations should be considered pathologic in a patient of a certain age and sex. We endorse further investigations on the clinical impact of age-adjusted and sex-adjusted upper reference limits in order to reduce underdiagnosis and overdiagnosis in an aging population.

## Figures and Tables

**Table 1:** Baseline characteristics of the study cohorts. Study cohort 1 (used for the calculation of age-specific and sex-specific upper reference limits) included subjects with preserved renal function only, whereas study cohort 2 (for the analysis of the relationship between hs-cTnT and kidney function) included an additional 208 individuals with an eGFR < 60 mL/min/1.73 m^2^. Age and laboratory parameters are presented as medians (interquartile range). Hs-cTnT: highly sensitive cardiac troponin T; HbA1c: hemoglobin A1c; NT-proBNP: amino-terminus pro B-type natriuretic peptide; eGFR: estimated glomerular filtration rate.

	Study Cohort 1	Study Cohort 2
female (n, %)	2,679 (49.4%)	2,789 (49.5%)
Age (years)	52.2 (45.0–62.3)	52.9 (45.2–63.3)
hs-cTnT (ng/L)	4.3 (3.0–6.0)	4.4 (3–6.2)
Creatinine (μmol/L)	77.0 (69.0–87.0)	78.0 (69–88)
HbA1c (mmol/mol)	34.4 ( 31.3–36.6)	34.4 (32.2–36.6)
NT-proBNP (ng/L)	48.0 (28.0–75.3)	49.1 (28.5–76.6)
eGFR (mL/min/1.73 m${}^{2}$)	87.1 (77.2–97.0)	86.4 (76.0–96.5)

**Table 2:** Age distribution, corresponding number of participants, age-adjusted median, and 99th percentile values of hs-cTnT (95% confidence interval) per group, divided by sex in a cardiac-healthy population in study cohort 1. Hs-cTnT: highly sensitive cardiac troponin T.

Age (Years)	Count (%)		Median hs-cTnT (ng/L)		99th Percentile hs-cTnT (ng/L)		*p*-Value
	Female	Male	Female	Male	Female	Male	
20–40	196 (47.6)	217 (52.4)	3.0	4.2	6.6 [5.0, 18.0]	13.7 [10.9, 16.5]	<0.001
41–50	992 (51.2)	945 (48.8)	3.0	4.5	7.4 [6.7, 9.7]	14.4 [12.1, 20.7]	<0.001
51–60	724 (50.3)	714 (49.7)	3.4	5.2	12.2 [8.8, 46.4]	14.7 [13.2, 20.0]	<0.001
61–70	522 (49.9)	525 (50.1)	4.5	6.4	12.5 [10.5, 18.2]	22.4 [18.4, 34.9]	<0.001
71–80	245 (41.3)	348 (58.7)	6.0	8.3	17.8 [14.2, 54.3]	30.1 [23.4, 40.0]	<0.001

**Table 3:** Median and 99th percentile hs-cTnT values in relation to serum creatinine level separated by sex in study cohort 2. The number of participants and median age (with interquartile range) is provided per creatinine group. Creatinine values are binned in increments of 50 μmol/L. 99th percentiles, and their 95% confidence intervals were provided in groups with at least 300 subjects. Increasing serum creatinine concentrations are associated with higher median hs-cTnT values and higher ages. Hs-cTnT: highly sensitive cardiac troponin T.

Creatinine (μmol/L)	Count		Median Age (Years)		Median Hs-cTnT (ng/L)		99th Perc. hs-cTnT (ng/L)	
	Female	Male	Female	Male	Female	Male	Female	Male
0–50	45	1	52 (48–62)	47 (47)	3.0	6.2	–	–
51–100	2723	2467	53 (45–62)	52 (45–63)	3.4	5.2	13.2 [12.0, 16.5]	20.2 [18.0, 23.1]
101–150	20	374	72 (61–75)	58 (47–70)	7.4	6.2	–	23.4 [19.8, 126.0]
151–250	1	5	79 (79)	76 (69–76)	23.7	18.9	–	–

**Table 4:** Median and 99th percentile hs-cTnT values in relation to renal function measured by eGFR and number of participants per group separated by sex, respectively, in study cohort 2. eGFR values are binned in increments of 30 mL/min/1.73 m${}^{2}$. The 99th percentiles and their 95% confidence intervals were given in groups with at least 300 subjects. Median hs-cTnT values increase with falling eGFR. Hs-cTnT: highly sensitive cardiac troponin T; eGFR: estimated glomerular filtration rate.

eGFR (mL/min/1.73 m${}^{2}$)	Count		Median Hs-cTnT (ng/L)		99th Perc. hs-cTnT (ng/L)	
	Female	Male	Female	Male	Female	Male
121–150	21	15	3.0	4.0	–	–
91–120	1018	1181	3.0	4.6	9.3 [8.0, 15.7]	16.6 [14.0, 20.7]
61–90	1640	1553	3.7	6.1	13.2 [12.0, 17.6]	22.1 [19.4, 26.2]
31–60	109	97	6.3	9.9	–	–
0–30	1	1	23.7	17.9	–	–

## Supplementary Materials

The following are available online at https://www.mdpi.com/article/10.3390/jcm10235508/s1, Table S1: Medians and 99th percentiles of highly sensitive cTnT in relation to age and sex, Figure S1: Distribution of eGFR values in different age bins, Figure S2: Study population, Figure S3: Floating medians and 99th percentiles of highly sensitive cTnT in relation to age and sex.

## Abbreviations

The following abbreviations are used in this manuscript:

AACC	Academy of the American Association for Clinical Chemistry
AMI	Acute myocardial infarction
CAD	Coronary artery disease
CKD-EPI	Chronic kidney disease epidemiology collaboration
ECLIA	Electrochemiluminescence immunoassay
eGFR	Estimated glomerular filtration rate
ESC	European Society of Cardiology
HbA1c	Hemoglobin A1c
hs-cTnT	Highly sensitive cardiac troponin T
IFCC	International Federation of Clinical Chemistry and Laboratory Medicine
NT-proBNP	Amino-terminus pro B-type natriuretic peptide
TINIA	Turbidimetric inhibition immuno assay
TRAPID-AMI	High Sensitivity Cardiac Troponin T assay for rapid Rule-out of Acute Myocardial Infarction Study

## References

[1] Collet J.P., Thiele H., Barbato E., Barthélémy O., Bauersachs J., Bhatt D.L., Dendale P., Dorobantu M., Edvardsen T., Folliguet T. (2020). ESC Guidelines for the management of acute coronary syndromes in patients presenting without persistent ST-segment elevation. Eur. Heart J..

[2] Amsterdam E.A., Wenger N.K., Brindis R.G., Casey D.E., Ganiats T.G., Holmes D.R., Jaffe A.S., Jneid H., Kelly R.F., Kontos M.C. (2014). AHA/ACC Guideline for the Management of Patients with Non-ST-Elevation Acute Coronary Syndromes: A report of the American College of Cardiology/American Heart Association Task Force on Practice Guidelines. J. Am. Coll. Cardiol..

[3] Thygesen K., Alpert J.S., Jaffe A.S., Chaitman B.R., Bax J.J., Morrow D.A., White H.D., Executive Group on behalf of the Joint European Society of Cardiology (ESC)/American College of Cardiology (ACC)/American Heart Association (AHA)/World Heart Federation (WHF) Task Force for the Universal Definition of Myocardial Infarction (2018). Fourth Universal Definition of Myocardial Infarction. J. Am. Coll. Cardiol..

[4] Giannitsis E., Kurz K., Hallermayer K., Jarausch J., Jaffe A.S., Katus H.A. (2010). Analytical validation of a high-sensitivity cardiac troponin T assay. Clin. Chem..

[5] Saenger A.K., Beyrau R., Braun S., Cooray R., Dolci A., Freidank H., Giannitsis E., Gustafson S., Handy B., Katus H. (2011). Multicenter analytical evaluation of a high-sensitivity troponin T assay. Clin. Chim. Acta.

[6] Gore M.O., Seliger S.L., Defilippi C.R., Nambi V., Christenson R.H., Hashim I.A., Hoogeveen R.C., Ayers C.R., Sun W., McGuire D.K. (2014). Age- and sex-dependent upper reference limits for the high-sensitivity cardiac troponin T assay. J. Am. Coll. Cardiol..

[7] Franzini M., Lorenzoni V., Masotti S., Prontera C., Chiappino D., Latta D.D., Daves M., Deluggi I., Zuin M., Ferrigno L. (2015). The calculation of the cardiac troponin T 99th percentile of the reference population is affected by age, gender, and population selection: A multicenter study in Italy. Clin. Chim. Acta.

[8] Kuster N., Monnier K., Baptista G., Dupuy A.M., Badiou S., Bargnoux A.S., Jeandel C., Cristol J.P. (2015). Estimation of age- and comorbidities-adjusted percentiles of high-sensitivity cardiac troponin T levels in the elderly. Clin. Chem. Lab. Med..

[9] Orlev A., Klempfner R., Rott D. (2018). Serum Cardiac Troponin T Levels in Asymptomatic Elderly Nursing Home Residents. Am. J. Med..

[10] Gaggin H.K., Dang P.V., Do L.D., deFilippi C.R., Christenson R.H., Lewandrowski E.L., Lewandrowski K.B., Truong B.Q., Pham V.Q., Vu V.H. (2014). Reference interval evaluation of high-sensitivity troponin T and N-terminal B-type natriuretic peptide in Vietnam and the US: The North South East West Trial. Clin. Chem..

[11] Kimenai D.M., Henry R.M., van der Kallen C.J., Dagnelie P.C., Schram M.T., Stehouwer C.D., van Suijlen J.D., Niens M., Bekers O., Sep S.J. (2016). Direct comparison of clinical decision limits for cardiac troponin T and I. Heart.

[12] Ungerer J.P.J., Tate J.R., Pretorius C.J. (2016). Discordance with 3 Cardiac Troponin I and T Assays: Implications for the 99th Percentile Cutoff. Clin. Chem..

[13] Aw T.-C., Phua S.-K., Lam C.-W., Wong M.-S. (2017). What are normal high-sensitivity troponin-T values in a large multi-ethnic Asian population?. Blood Heart Circ..

[14] Giannitsis E., Mueller-Hennessen M., Zeller T., Schuebler A., Aurich M., Biener M., Vafaie M., Stoyanov K.M., Ochs M., Riffel J. (2020). Gender-specific reference values for high-sensitivity cardiac troponin T and I in well-phenotyped healthy individuals and validity of high-sensitivity assay designation. Clin. Biochem..

[15] Sandstede J., Lipke C., Beer M., Hofmann S., Pabst T., Kenn W., Neubauer S., Hahn D. (2000). Age- and gender-specific differences in left and right ventricular cardiac function and mass determined by cine magnetic resonance imaging. Eur. Radiol..

[16] Wingren C.J., Ottosson A. (2015). Postmortem heart weight modelled using piecewise linear regression in 27,645 medicolegal autopsy cases. Forensic. Sci. Int..

[17] Aksoy N., Ozer O., Sari I., Sucu M., Aksoy M., Geyikli I. (2009). Contribution of renal function impairment to unexplained troponin T elevations in congestive heart failure. Ren. Fail..

[18] Chotivanawan T., Krittayaphong R. (2012). Normal range of serum highly-sensitive troponin-T in patients with chronic kidney disease stage 3–5. J Med. Assoc. Thai..

[19] Pfortmueller C.A., Funk G.C., Marti G., Leichtle A.B., Fiedler G.M., Schwarz C., Exadaktylos A.K., Lindner G. (2013). Diagnostic performance of high-sensitive troponin T in patients with renal insufficiency. Am. J. Cardiol..

[20] Dubin R.F., Li Y., He J., Jaar B.G., Kallem R., Lash J.P., Makos G., Rosas S.E., Soliman E.Z., Townsend R.R. (2013). Predictors of high sensitivity cardiac troponin T in chronic kidney disease patients: A cross-sectional study in the chronic renal insufficiency cohort (CRIC). BMC Nephrol..

[21] Chung J.Z.Y., Dallas Jones G.R. (2015). Effect of renal function on serum cardiac troponin T–Population and individual effects. Clin. Biochem..

[22] Twerenbold R., Wildi K., Jaeger C., Gimenez M.R., Reiter M., Reichlin T., Walukiewicz A., Gugala M., Krivoshei L., Marti N. (2015). Optimal Cutoff Levels of More Sensitive Cardiac Troponin Assays for the Early Diagnosis of Myocardial Infarction in Patients with Renal Dysfunction. Circulation.

[23] Chesnaye N.C., Szummer K., Bárány P., Heimbürger O., Magin H., Almquist T., Uhlin F., Dekker F.W., Wanner C., Jager K.J. (2019). Association between Renal Function and Troponin T Over Time in Stable Chronic Kidney Disease Patients. J. Am. Heart Assoc..

[24] Twerenbold R., Badertscher P., Boeddinghaus J., Nestelberger T., Wildi K., Puelacher C., Sabti Z., Rubini Gimenez M., Tschirky S., du Fay de Lavallaz J. (2018). 0/1-Hour Triage Algorithm for Myocardial Infarction in Patients with Renal Dysfunction. Circulation.

[25] Omar A.S., Mahmoud K., Hanoura S., Osman H., Sivadasan P., Sudarsanan S., Shouman Y., Singh R., AlKhulaifi A. (2017). Acute kidney injury induces high-sensitivity troponin measurement changes after cardiac surgery. BMC Anesthesiol..

[26] Loeffler M., Engel C., Ahnert P., Alfermann D., Arelin K., Baber R., Beutner F., Binder H., Brähler E., Burkhardt R. (2015). The LIFE-Adult-Study: Objectives and design of a population-based cohort study with 10,000 deeply phenotyped adults in Germany. BMC Public Health.

[27] Wu A.H.B., Christenson R.H., Greene D.N., Jaffe A.S., Kavsak P.A., Ordonez-Llanos J., Apple F.S. (2018). Clinical Laboratory Practice Recommendations for the Use of Cardiac Troponin in Acute Coronary Syndrome: Expert Opinion from the Academy of the American Association for Clinical Chemistry and the Task Force on Clinical Applications of Cardiac Bio-Markers of the International Federation of Clinical Chemistry and Laboratory Medicine. Clin. Chem..

[28] Levey A.S., Stevens L.A., Schmid C.H., Zhang Y.L., Castro A.F., Feldman H.I., Kusek J.W., Eggers P., Van Lente F., Greene T. (2009). A new equation to estimate glomerular filtration rate. Ann. Intern. Med..

[29] Inker L.A., Eneanya N.D., Coresh J., Tighiouart H., Wang D., Sang Y., Crews D.C., Doria A., Estrella M.M., Froissart M. (2021). New Creatinine- and Cystatin C–Based Equations to Estimate GFR without Race. N. Engl. J. Med..

[30] Harris C.R., Millman K.J., van der Walt S.J., Gommers R., Virtanen P., Cournapeau D., Wieser E., Taylor J., Berg S., Smith N.J. (2020). Array programming with NumPy. Nature.

[31] GitHub: QuantileCI. https://github.com/hoehleatsu/quantileCI.

[32] R Cor Team (2014). A Language and Environment For Statistical Computing.

[33] Villanueva R.A.M., Chen Z.J. (2016). ggplot2: Elegant Graphics for Data Analysis.

[34] Adler D., Murdoch D. (2017). Rgl: 3D Visualization Using OpenGL. https://www.researchgate.net/publication/318392813_Rgl_3D_Visualization_Using_OpenGL.

[35] van Rossum G., Drake F.L. (2009). Python 3 Reference Manual.

[36] Shah A.S.V., Griffiths M., Lee K.K., McAllister D.A., Hunter A.L., Ferry A.V., Cruikshank A., Reid A., Stoddart M., Strachan F. (2015). High sensitivity cardiac troponin and the under-diagnosis of myocardial infarction in women: Prospective cohort study. BMJ.

[37] Eggers K.M., Lindahl B. (2017). Impact of Sex on Cardiac Troponin Concentrations—A Critical Appraisal. Clin. Chem..

[38] Mueller-Hennessen M., Lindahl B., Giannitsis E., Biener M., Vafaie M., Defilippi C.R., Christ M., Santalo-Bel M., Panteghini M., Plebani M. (2016). Diagnostic and prognostic implications using age- and gender-specific cut-offs for high-sensitivity cardiac troponin T—Sub-analysis from the TRAPID-AMI study. Int. J. Cardiol..

[39] Rubini Giménez M., Twerenbold R., Boeddinghaus J., Nestelberger T., Puelacher C., Hillinger P., Wildi K., Jaeger C., Grimm K., Heitzelmann K.F. (2016). Clinical Effect of Sex-Specific Cutoff Values of High-Sensitivity Cardiac Troponin T in Suspected Myocardial Infarction. JAMA Cardiol..

[40] Enzenbach C., Wicklein B., Wirkner K., Loeffler M. (2019). Evaluating selection bias in a population-based cohort study with low baseline participation: The LIFE-Adult-Study. BMC Med. Res. Methodol..

[41] (2013). KDIGO 2012 Clinical Practice Guideline for the Evaluation and Management of Chronic Kidney Disease. Kidney Int. Suppl..

