# Supplementary material for: Revisited Upper Reference Limits for Highly Sensitive Cardiac Troponin T in Relation to Age, Sex, and Renal Function
Source: J Clin Med. 2021 Nov 25;10(23):5508. doi: 10.3390/jcm10235508 (PMC8658212; doi:10.3390/jcm10235508)
Supplement: Supplementary file 1 [file jcm-10-05508-s001.zip › jcm-1400916.pdf]

Revisited Upper Reference Limits for Highly Sensitive Cardiac Troponin T in Relation to Age, Sex, and Renal Function - Online Supplement

Christiane Gärtner, Romy Langhammer, Maria Schmidt, Martin Federbusch, Kerstin Wirkner, Markus Löffler, Berend Isermann, Ulrich Laufs, Rolf Wachter, and Thorsten Kaiser

Table S1: Age distribution, corresponding number of participants, age-adjusted median and 99th percentile values of hs-cTnT [95% confidence interval] per group, divided by sex in a cardiac-healthy population. Additionally to study cohort 1, all subjects with an eGFR < 90 ml/min/1.73 m^2 were excluded. Hs-cTnT: highly sensitive cardiac troponin T.

**Age (years)**	**Count (%)**		**Median hs-cTnT (ng/l)**		**99th percentile hs-cTnT (ng/l)**		**p-value**
	female	male	female	male	female	male	
20–40	154 (48.3)	165 (51.7)	3.0	4.3	6.0 [5.0, 8.2]	13.6 [8.3, 16.5]	< 0.001
41–50	504 (43.9)	590 (56.1)	3.0	4.3	7.4 [6.1, 14.3]	13.9 [11.6, 25.0]	< 0.001
51–60	258 (45.3)	312 (64.7)	3.2	4.9	12.7 [8.2, 73.9]	13.7 [13.0, 20.4]	< 0.001
61–70	113 (48.5)	120 (51.5)	4.0	5.5	12.8 [10.5, 26.7]	22.4 [19.5, 126.2]	< 0.001
71-80	10 (52.6)	9 (47.4)	4.0	5.5	6.0 [5.2, 6.1]	29.3 [23.1, 29.9]	–

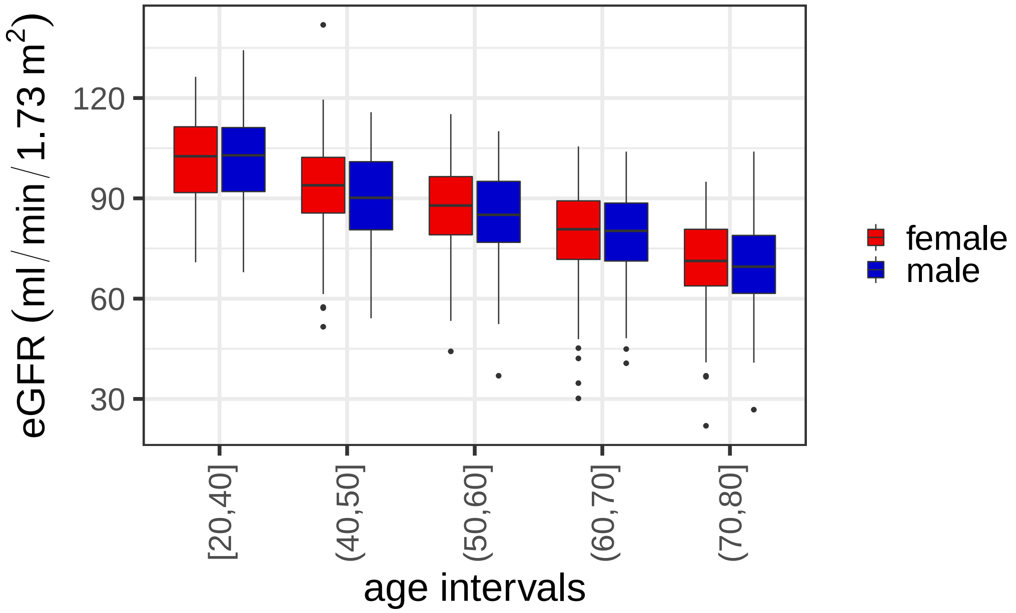

Figure S1: Distribution of eGFR values in different age bins. Higher age correlates to lower median eGFR values.

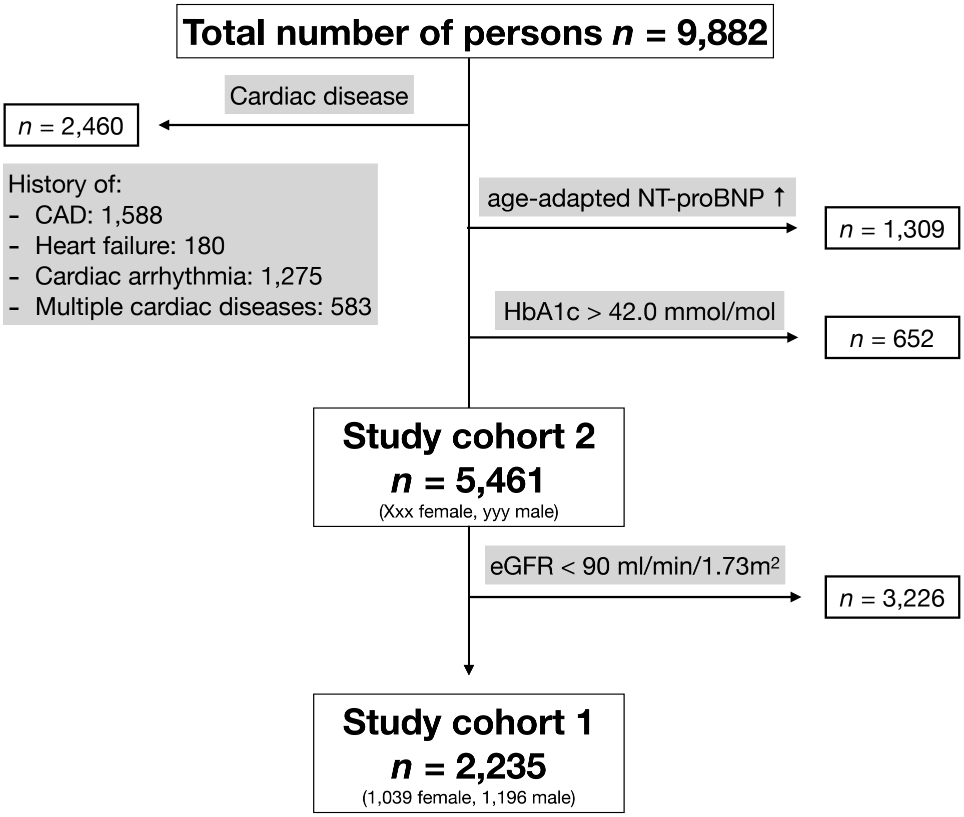

Figure S2: Study population: of 9,882 adults, 2,460 were excluded due to cardiac diseases and 1,961 due to abnormal NT-proBNP or HbA1c values. For the calculation of age- and sex-adjusted hs-cTnT values, additionally, 3,226 participants with an impaired eGFR (< 90 ml/min/1.73 m^2) were excluded (study cohort 1). CAD: coronary artery disease, HbA1c: hemoglobin A1c, NT-proBNP: amino-terminus pro B-type natriuretic peptide, eGFR: estimated glomerular filtration rate.

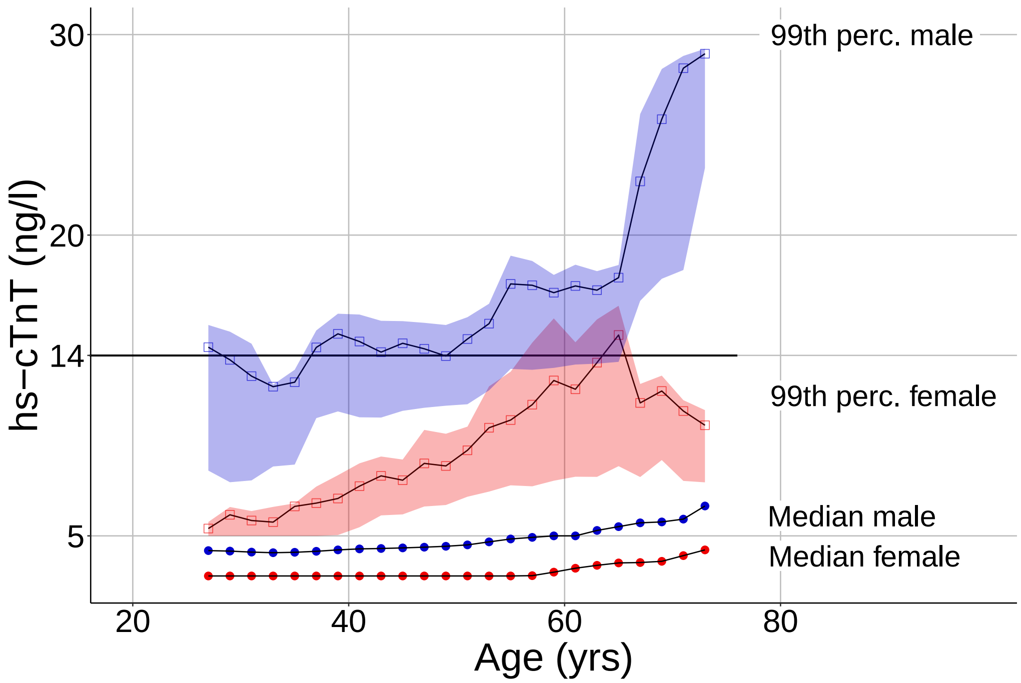

Figure S3: Age-specific floating median (circles) and 99th percentile (squares) hs-cTnT values in females (red) and males (blue). The ranges of 97th–99.4th hs-cTnT percentiles (smoothed with a window size of +/- 10 years per point) are highlighted in light red (females) and light blue (males). Hs-cTnT: highly sensitive troponin T.
